# Effects of Dietary Medium-Chain Triglyceride Supplementation on the Serum Metabolome of Young Adult and Senior Canines

**DOI:** 10.3390/ani14243577

**Published:** 2024-12-11

**Authors:** Yuanlong Pan, Miriam Sindelar, Ethan Stancliffe, Leah P. Shriver, Rondo P. Middleton, Gary J. Patti

**Affiliations:** 1 Nestlé Purina Research, St. Louis, MO 63164, USA; rondo.middleton@rd.nestle.com; 2 Department of Chemistry, Washington University in St. Louis, St. Louis, MO 63130, USA; miriam.sindelar@wustl.edu (M.S.); estancliffe@wustl.edu (E.S.); sleah@wustl.edu (L.P.S.); 3 Center for Mass Spectrometry and Metabolic Tracing, Washington University in St. Louis, St. Louis, MO 63130, USA; 4 Center for Human Nutrition, Department of Medicine, Washington University in St. Louis, St. Louis, MO 63130, USA

**Keywords:** dog, medium-chain triglycerides (MCTs), metabolism, metabolomics, amino acids, lipids, ketone bodies

## Abstract

Canine cognitive dysfunction syndrome is a progressive condition that is characterized by deficits in memory and learning, behavioral changes, and decreased response to stimuli. This disorder, which currently lacks effective therapies, leads to a reduced quality of life for both dogs and their owners. Diets rich in medium-chain triglycerides (MCTs) have been shown to positively impact brain health; however, their effects on systemic metabolism have been incompletely characterized. This study used an untargeted metabolomics platform to profile alterations in the serum metabolome of young adult and senior canines fed a diet containing MCTs. MCT feeding induced alterations in the levels of circulating complex lipids (including triacylglycerides, glycerophospholipids, and cholesterol esters), ketone bodies, glutamine, and some branched-chain and aromatic amino acids. Collectively, the results suggest that MCT supplementation rewires global metabolism and could potentially improve health beyond cognitive function.

## Abstract (plain language summary)

Canine cognitive dysfunction syndrome is a progressive disorder that is characterized by impaired memory and learning as well as behavioral changes. This disorder adversely affects the quality of life of both dogs and their owners. Diets rich in medium-chain triglycerides (MCTs) have been shown to significantly improve cognitive function in senior dogs; however, the effects of dietary MCTs on systemic metabolism have not been investigated. The current study used an untargeted metabolomics platform to evaluate the impact of dietary MCTs on the serum metabolome of young adult and senior dogs. The data show that MCT feeding induces changes in the levels of circulating complex lipids, ketone bodies, alanine, glutamine, and some branched-chain and aromatic amino acids. The results suggest that dietary MCTs rewire global metabolism and could potentially improve health beyond cognitive function.

## 1. Introduction

In dogs, brain aging may lead to cognitive decline and cognitive dysfunction syndrome (CDS). CDS is characterized by disorientation, reduced social interactions, increased anxiety, a disrupted sleep/wake cycle, inappropriate barking and soiling, and other abnormal changes in behavior [1,2]. As a result, cognitive impairment and CDS can adversely affect the quality of life of both pets and pet owners. Some research has focused on developing nutritional interventions to delay brain aging, cognitive decline, and symptoms of CDS in dogs [3,4]. One nutritional approach is to address the reduced metabolism of glucose in the brain [4].

The decreased metabolism of glucose in the brain is a common feature of aging and has been reported in healthy senior rodents [5], healthy senior dogs [6], healthy senior monkeys [7], and healthy senior people [8]. Studies in rats and dogs indicate that cerebral glucose metabolism is already reduced in middle-aged animals [5,6]. Age-related brain pathology also impacts the utilization of glucose. Cerebral glucose metabolism was significantly reduced in patients with Alzheimer’s disease (AD) compared to healthy age-matched control subjects [9]. Of note, progression from mild cognitive impairment to AD was associated with further declines in cerebral glucose metabolism [10]. These data suggest that a reduction in cerebral glucose metabolism occurs in all animals and may partially contribute to the development, progression, and symptoms of cognitive decline, cognitive impairment, and AD.

A strategy to improve cognitive function in senior dogs is to compensate for reduced cerebral glucose metabolism by providing alternative energy sources, such as ketone bodies, to the brain [4]. Ketone bodies are mainly produced by the liver from body fat and utilized by extrahepatic tissues. The circulating concentration of ketone bodies can be increased by dietary supplementation with medium-chain triglycerides (MCTs), which are rapidly converted to ketone bodies by the liver and, to a lesser extent, by astrocytes in the brain [11]. Ketone bodies can then be used by neurons as an alternative energy source to alleviate the deficits in glucose metabolism [12]. This increases energy availability in the brain and helps to maintain or improve brain function. The MCT intervention model described here is supported by evidence that MCT supplementation improves cognitive performance in subjects with AD [13]. Knottnerus et al. reported that, when administered as a single energy source in healthy people, about 42% of MCTs were metabolized to CO_2_. The oxidation of MCTs was increased to 62% when MCTs were consumed in combination with carbohydrates and proteins in the diet. Under both conditions, less than 1% of ^13^C-labeled MCT carbon was incorporated into long-chain fatty acids in plasma [14]. Although the relative MCT oxidation rate was higher in combination with carbohydrates and protein, quantitatively, more MCTs were oxidized when they were administered as an isocaloric dose of only MCTs. MCTs significantly improved cognitive ability in APOE4^-/-^ patients with mild to moderate AD [14]. The beneficial effects of MCTs might be related to their impact on the metabolism of lysophosphatidylcholines (LPCs), oleate, linoleate, and palmitate along with the ketogenic effects. Specifically, the concentrations of total cholesterol (TC), high-density lipoprotein cholesterol (HDL-C), β-hydroxybutyrate, and acetoacetate were significantly higher in the MCT group compared to the placebo group. LPC (16:0), LPC (18:0), LPC (18:1), LPC (20:2), and LPC (22:5) were significantly increased, and LPC (18:0), palmitate, linoleate, and oleate were significantly decreased after the MCT intervention [15].

Untargeted metabolomics and lipidomics are powerful technologies to dissect alterations in systemic metabolism in response to diet. When performing untargeted metabolomics and lipidomics with liquid chromatography/mass spectrometry (LC/MS), hundreds to thousands of chemicals can be profiled spanning amino acids, vitamins, lipids, and energy metabolites. LC/MS is able to measure compounds that are directly derived from the diet as well as those produced by the body in response to dietary intervention [16,17]. To date, there have been few studies in canines utilizing metabolomics to assess dietary responses. Changes in the serum metabolome were observed in animals fed a high-protein diet, where metabolites associated with inflammation and kidney dysfunction were increased with a greater protein intake [18]. At this time, however, no study has been conducted in dogs to determine how dietary MCTs influence whole-body metabolism. The objective of this study was to use untargeted metabolomics and lipidomics to profile changes in the serum of dogs administered an MCT-enriched diet.

## 2. Methods

### 2.1. Animals and Housing Conditions

The study protocol was approved by the Nestlé Purina Institutional Animal Care and Use Committee. A total of 20 beagles (10 young adults and 10 senior) and 19 Labrador retrievers (9 young adults and 10 senior) with body condition scores (BCSs) between 4 and 6 on a 9-point BCS scale [19] were recruited for this study. Young adult dogs were between 1 and 6 years of age, while senior dogs were 7 years and older. Table 1 and Table 2 summarize the demographics of the dogs in the study. Each dog’s maintenance energy requirement (MER) was estimated by a standard calculation (139 × (BW in kg)^0.75^) [20].

Dogs were housed in a climate-controlled facility with indoor/outdoor access. Indoor areas had both natural and artificial lighting, along with air conditioning/heating and fans for air circulation. The indoor kennel size was 50” × 108” (~37 square feet), and the outdoor kennel size was 52” × 180” (~65 square feet). Dogs had access to elevated beds, environmental enrichment (toys), and an automated drinking system with ad libitum access to fresh water. Dogs were pair-housed with access to two individual indoor runs and a shared outdoor run at all times, except during feeding, when dogs were separated into individual indoor runs to allow for the accurate tracking of food consumption. Indoor/outdoor runs were cleaned once daily. Feeding bowls were cleaned every day. Dogs had daily contact with the care staff and their kennelmate. Dogs were taken out for walks, play yard sessions, and/or socialization events together with other dogs from their established play groups daily.

### 2.2. Diets

The control diet was a super premium adult dry dog food. The test diet was formulated by replacing 5.5% beef tallow with 5.5% MCT oil (C8:0, C10:0) in the control diet. The ingredients and compositions of the diets are summarized in Table 3. Both diets were isocaloric and had identical ingredients, except 5.5% MCT in the MCT diet and 5.5% tallow in the control. To confirm the presence of MCT species in the diet, the compositions and relative abundances of dietary MCTs and MCT oil used to make the test diet were measured by LC/MS and are shown in Table 4 [21]. The abundance of each MCT species is expressed as the peak area detected with LC/MS.

### 2.3. Randomization

Within each breed and age group (young/senior), dogs were randomized into two diet groups with 5 dogs per group, with the exception of young Labrador retrievers based on estimated MERs, body weight, gender, and age. One group had four young Labrador retrievers and the other group had five young Labrador retrievers.

### 2.4. Feeding Protocol

**Figure 1:**
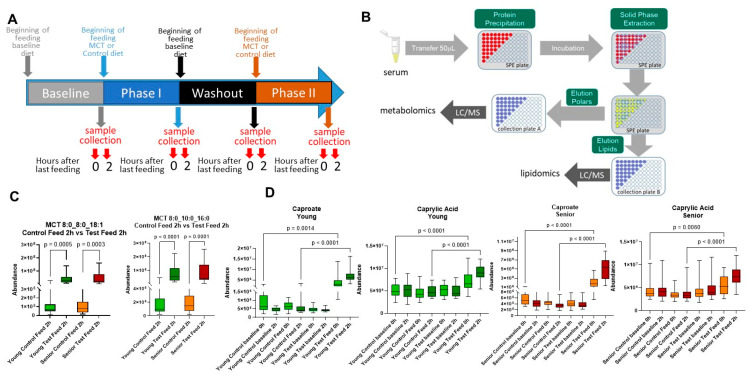
MCT diet influences medium-chain fatty acid availability in circulation. (**A**) Schematic of the cross-over study design. (**B**) Solid-phase extraction protocol for the isolation of polar metabolites and lipids from serum samples. (**C**) Relative abundance of MCT species with medium-chain fatty acids and long-chain fatty acids in circulation before and after MCT diet feeding in young adult (green) and senior (red) dogs. (**D**) Relative abundance of serum medium-chain fatty acids during the control and MCT diet feeding in young adult and senior dogs. The values of each box represent the 25th and 75th percentiles, and whiskers are maximum and minimum values. *p* statistics are from paired Student’s *t*-tests or ANOVA with Šídák’s correction.

Dogs were fed 100% of their MERs for 20 weeks through the study to maintain their body weight (i.e., less than 5% change in body weight during the feeding phases). The feeding study proceeded with the feeding phases described below. In this cross-over study, adult dogs were fed two different diets to assess the impact of MCT supplementation on the levels of circulating metabolites and lipids (study workflow prior to the metabolomics and lipidomics analysis is shown in Figure 1A). The goals of the study were to track alterations in metabolites and lipids in response to the diet across participants and determine whether there were differences in metabolic responses between young and senior dogs. A total of 39 dogs in the study were randomized into two groups and fed both control and test diets separated by a washout period. One group was first fed the control diet, followed by a washout period then fed the test diet. The second group was fed the test diet first, followed by a washout period and then the control diet.

#### 2.4.1. The Pretest Phase

The dogs were fed the control diet at 100% of their estimated MERs for 5 weeks to stabilize their body weight (i.e., less than 5% change in three weeks) by adjusting their food intake and to standardize their metabolomics profiles. At the end of the phase, blood samples were collected at 0 (fasting blood sample right before feeding) and 2 h after feeding with serum Separator tubes (456073P, Greiner Bio-One, Monroe, NC, USA) for metabolomics and lipidomics analyses as the baseline samples for both the control and test dogs.

#### 2.4.2. The Cross-Over Phase One

During this phase, using baseline randomization, dogs were fed either the control diet or the MCT diet. Dogs were fed the diets at 100% of their adjusted MERs for 5 weeks in the morning. At the end of this phase, blood samples were collected at 0 (fasting blood sample right before feeding) and 2 h after feeding with serum Separator tubes (456073P, Greiner Bio-One, Monroe, NC, USA) for metabolomics and lipidomics analyses.

#### 2.4.3. The Wash-Out Phase

During this phase, all dogs were fed the control diet at 100% of their adjusted MERs for 5 weeks in the morning. At the end of the phase, blood samples were collected at 0 (fasting blood sample right before feeding) and 2 h after feeding with serum Separator tubes (456073P, Greiner Bio-One, Monroe, NC) for metabolomics and lipidomics analyses.

#### 2.4.4. The Cross-Over Phase Two

During this phase, the dogs fed the MCT diet during the cross-over phase one were switched to the control diet, and the dogs fed the control diet during the cross-over phase one were switched to the MCT diet. Dogs were fed the diets at 100% of their adjusted MERs in the morning for 5 weeks. At the end of the phase, blood samples were collected at 0 (fasting blood sample right before feeding) and 2 h after feeding with serum Separator tubes (456073P, Greiner Bio-One, Monroe, NC) for metabolomics and lipidomics analyses.

### 2.5. Sample Preparation for Metabolomics and Lipidomics

Metabolomics was performed as described previously [23]. Serum, which had been stored at −80 °C upon collection, was thawed on ice. A 50 µL aliquot was transferred onto a solid-phase extraction (SPE) system CAPTIVA-EMR Lipid 96-wellplate (Agilent Technologies, Santa Clara, CA, USA) before the addition of 250 µL of acetonitrile containing 1% formic acid (*v*/*v*), or alternatively methanol:ethanol 1:1 (*v*/*v*), and 10 µM internal standards consisting of uniformly ^13^C- and ^15^N-labeled amino acids from Cambridge Isotope Laboratories, Inc., (Tewksbury, MA, USA). The samples were mixed for 1 min at 360 rpm on an orbital shaker at room temperature, followed by a 10 min incubation period at 4 °C, and the addition of 200 µL 80% acetonitrile in water (*v*/*v*) to the samples. The samples were then mixed on an orbital shaker (360 rpm) for an additional 10 min at room temperature to complete mixing and protein precipitation. Next, the samples were eluted into a 96-deepwell collection plate by centrifugation (10 min, 57× *g*, 4 °C followed by 2 min, 1000× *g*, 4 °C). The SPE plates were then washed twice with 500 µL 80% acetonitrile in water (*v*/*v*). Lipids still bound to the SPE material were then released into a second elution plate, in two elution steps by applying 2 × 500 µL 1:1 methyl-tert-butyl ether:methanol (*v*/*v*) onto the SPE cartridge and centrifuging at 4 °C for 2 min at 1000× *g*. The combined eluates were dried under a stream of nitrogen (Biotage SPE Dry Evaporation System, Biotage, Uppsala, Sweden) at room temperature and reconstituted with 100 µL 1:1 2-propanol:methanol (*v*/*v*) prior to LC/MS analysis.

### 2.6. LC/MS Analysis of Polar Metabolites

An aliquot of 2 µL of polar metabolite extract was analyzed by using an Agilent 1290 Infinity II liquid chromatography (LC) system coupled to an Agilent 6540 Quadrupole-Time-of-Flight (Q-TOF) mass spectrometer (Agilent, Santa Clara, CA, USA). Metabolites were separated on a SeQuant^®^ ZIC^®^-pHILIC column (100 × 2.1 mm, 5 µm, polymer, Merck-Millipore, Burlington, MA, USA). A ZIC^®^-pHILIC guard column (2.1 mm × 20 mm, 5 µm) was used. The column compartment temperature was maintained at 40 °C and the flow rate was set to 250 µL·min^−1^. The mobile phases consisted of A: 95% water, 5% acetonitrile, 20 mM ammonium bicarbonate, 0.1% ammonium hydroxide solution (25% ammonia in water), and 2.5 µM medronic acid, and B: 95% acetonitrile, 5% water, and 2.5 µM medronic acid. The following linear gradient was applied: 0 to 1 min, 90% B; 1 to 12 min, 35% B; 12 to 12.5 min, 25% B; 12.5 to 14.5 min, 25% B; 14.5 to 15 min, 90% B followed by a re-equilibration phase of 4 min at 400 µL·min^−1^ and 2 min at 250 µL·min^−1^. Metabolites were detected in positive and negative ion mode with the following source parameters: gas temperature of 200 °C, drying gas flow of 10 L·min^−1^, nebulizer pressure of 44 psi, sheath gas temperature of 300 °C, sheath gas flow of 12 L·min^−1^, VCap of 3000 V, nozzle voltage of 2000 V, Fragmentor of 100 V, Skimmer of 65 V and Oct 1 RF Vpp of 750 V, and *m*/*z* range of 50–1700. Data were acquired under continuous reference mass correction at *m/z* 121.0509 and 922.0890 (positive ion mode) and *m*/*z* 119.036 and 966.0007 (negative ion mode). Samples were randomized before analysis. In addition, a quality-control sample was injected after every 12th research sample to monitor the signal stability of the instrument. Iterative MS/MS fragmentation data were acquired on a pooled plasma sample utilizing the MassHunter Acquisition Software (Version 10.1.48, Agilent Technologies). MS/MS were acquired at a scan rate of 3 spectra/s with different intensity thresholds and collision energies of 10, 20, and 40 V to increase identification rates.

To maximize the number of compounds identified, MS/MS spectra for polar metabolites were also acquired on an Orbitrap ID-X Tribrid mass spectrometer (Thermo Scientific, Waltham, MA, USA). The same chromatographic method as described above was applied on a Vanquish Horizon UHPLC system. Samples were analyzed in both positive and negative modes with a spray voltage of 3.5 and 2.8 kV, respectively. The RF lens value was 35%. Data were acquired in data-dependent acquisition (DDA) mode using AcquireX (Thermo Fisher Scientific, Waltham, MA) with a mass range of 67–900 *m*/*z*. The MS/MS isolation window was set to 1.5 Da and MS/MS scans were acquired at 15 K resolution. This workflow was performed on a pooled sample with different collision energies in the range of 20 to 50% for HCD and 30% for CID to maximize identifications.

### 2.7. LC/MS Analysis of Lipid Metabolites

An aliquot of 2 µL of lipid extract was subjected to LC/MS analysis by using an Agilent 1290 Infinity II LC system coupled to an Agilent 6545 Q-TOF mass spectrometer. Lipids were separated on an Acquity UPLC^®^ HSS T3 column (2.1 × 150 mm, 1.8 µm) with an Acquity UPLC^®^ HSS T3 VanGuard Pre-Column (2.1 × 5 mm, 1.8 µm, Waters Corp., Milford, MA). The column was maintained at a temperature of 60 °C. A flow rate of 250 µL·min^−1^ was applied. The mobile phases consisted of A: 60% acetonitrile, 40% water, 0.1% formic acid, 10 mM ammonium formate, and 2.5 µM medronic acid, and B: 90% 2-propanol, 10% acetonitrile, 0.1% formic acid, and 10 mM ammonium formate (dissolved in 1 mL water). The following linear gradient was used: 0–2 min, 30% B; 2–17 min, 75% B; 17–20 min, 85% B; 20–23 min, 100% B; 23–26 min, 100% B; 26–27 min, 30% B followed by a re-equilibration phase of 4 min.

Lipids were detected in positive and negative ion modes with the following source parameters: gas temperature of 250 °C, drying gas flow of 11 L·min^−1^, nebulizer pressure of 35 psi, sheath gas temperature of 300 °C, sheath gas flow of 12 L·min^−1^, VCap of 3000 V, nozzle voltage of 500 V, Fragmentor of 160 V, Skimmer of 65 V and Oct 1 RF Vpp of 750 V, and *m/z* range of 50–1700. Data were acquired under continuous reference mass correction at *m/z* 121.0509 and 922.0890 (positive ion mode) and *m/z* 119.036 and 966.0007 (negative ion mode). Samples were randomized before analysis. In addition, a quality control sample was injected after every 12th research sample to monitor signal stability of the instrument. Iterative MS/MS data were acquired on a pooled plasma sample, as described above for polar metabolites.

### 2.8. Compound Identification and Data Processing

Compound identifications were supported by matching the retention time, accurate mass, and MS/MS fragmentation data to online MS/MS libraries and our in-house library created from authentic reference standards. The MS/MS libraries used were as follows: Human Metabolome Database (HMDB, https://hmdb.ca, accessed on 23 November 2024), Mass Bank of North America (MoNA, https://mona.fiehnlab.ucdavis.edu/, accessed on 23 November 2024), and mzCloud (https://mzcloud.org, accessed on 23 November 2024). Lipid iterative MS/MS data were annotated with the Agilent Lipid Annotator software tool (Version 1.0). For quantitative profiling, all data files were then analyzed in Skyline (Version 20.1.0.155), as previously described [24].

### 2.9. Statistical Analysis

Differences in the levels of circulating metabolites and lipids were analyzed between senior and young adult animals by using either a paired Student’s *t*-test or a two-way ANOVA with Šídák’s correction for multiple comparisons’ statistical testing carried out by using GraphPad Prism v.10.1.2.

## 3. Results

Serum was collected from dogs and polar metabolites (e.g., amino acids, ketoacids, nucleotides, etc.) and lipids from serum were analyzed (Figure 1B). To confirm that animals received the MCT diet, the levels of circulating MCTs were measured. Animals administered the MCT diet showed increased levels of MCT species (Figure 1C) as well as medium-chain fatty acids, capric acid (C10:0), and caprylic acid (C8:0) (Figure 1D) compared to the control diet period. This suggests that feeding MCTs increases both circulating MCT levels as well as free fatty acids derived from the lipolysis of MCT species.

### 3.1. MCT Feeding Impacts Ketogenesis and Composition of Complex Lipid Species

**Figure 2:**
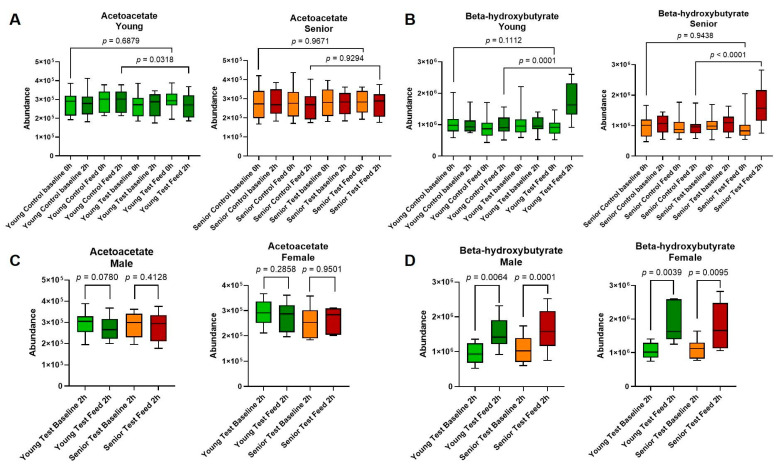
(**A**) β-hydroxybutyrate levels were increased in the serum after MCT feeding. Relative abundance of ketones, acetotacetate (**A**), and β-hydroxybutyrate (**B**), in the serum during control and MCT diet feeding in young adult and senior dogs. Relative abundance of circulating acetoacetate (**C**) and β-hydroxybutyrate (**D**) in young adults versus old dogs across males and females. Values of boxes represent the 25th and 75th percentiles and whiskers are maximum and minimum values. Results from young adults (green) and senior (red) animals are shown and *p* statistics are ANOVA with and Šídák’s correction.

Increased levels of MCTs have the potential to impact systemic metabolism by stimulating the production of ketone bodies through their lipolysis and the subsequent transformation of the medium-chain fatty acids into β-hydroxybutyrate (BHB), acetoacetate, and acetate by the liver [25]. Ketone bodies not only serve as an energy source for tissues but are also potent signaling molecules that could alter systemic metabolism [26]. First, analysis was conducted to investigate whether feeding MCTs would lead to increased levels of circulating ketone bodies by examining the plasma levels of BHB and acetoacetate. While BHB increased after feeding the test diet, the levels of acetoacetate were similar between the test and control diets. This effect was consistent in both young adult and senior dogs and was not dependent on the breed or sex of the animals (Figure 2A–D and Supplementary Materials Figure S1).

**Figure 3:**
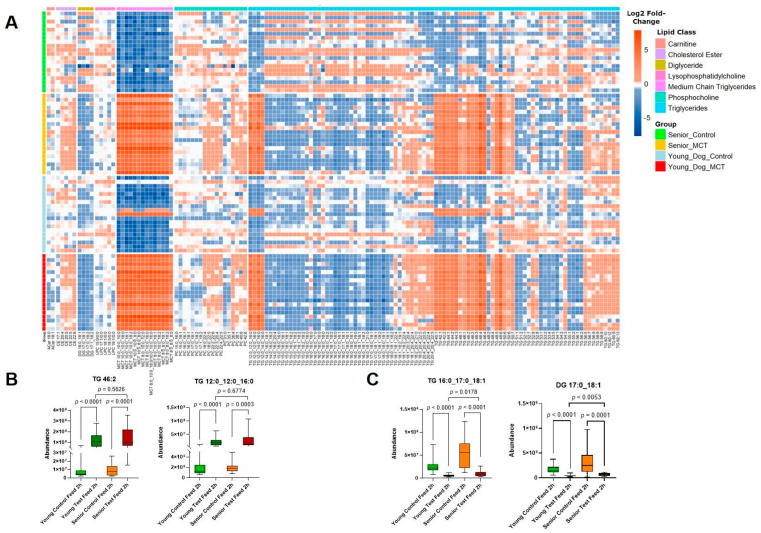
MCT feeding impacts the composition of complex lipid species. (**A**) Heatmap of lipids altered by MCT diet in young adult and senior animals. (**B**) Abundance of two representative TG species containing medium-chain fatty acids during control diet and MCT diet feeding in young adult and senior dogs. (**C**) Abundance of two representative TGs containing long-chain fatty acids (>C16) in young adult and senior animals. Values of boxes represent the 25th and 75th percentiles, and whiskers are maximum and minimum values. Results from young (green) and senior (red) animals are shown and *p* statistics are from paired Student’s *t*-tests or ANOVA with Šídák’s correction.

Leveraging our lipidomics data, we then turned our attention to various classes of lipid species. In the participant animals, 315 lipids were identified in serum. Notably, 180 were found to be significantly changed between the control and MCT diets in the young adult dogs and 155 species in the senior dogs (Figure 3A). Of those, 70 species were shared between the age groups, with 40 unique species for the young adult dogs and 15 for the senior dogs. Major lipid classes, such as glycerophospholipids, cholesterol esters (CEs), and triglycerides (TGs), were all impacted by the MCT diet. Additionally, complex lipids were biased to contain fatty acid chains from C8 to C14, suggesting that the fatty acids present from MCTs in the diet were reincorporated into storage and membrane lipids. Where the chain lengths were able to be determined, C12:0 and C14:0 fatty acids were identified as part of the compound. We point out that C12:0 and C14:0 fatty acids were not identified in the food samples analyzed at the start of the study (Table 4). Conversely, the TGs with more than 50 carbons (TG C50) decreased in the test diet. Where it was possible to identify FA chain lengths, those with FA chain lengths of C16, C17, and C18 all decreased as did diacylglycerides (DGs) with similar FA chain lengths (Figure 3B–C). Some alterations in the circulating lipid species showed breed-specific changes. Several unsaturated TG species decreased in young adult versus senior beagles, but not Labrador retrievers (Supplementary Materials Figure S2).

### 3.2. MCT Diet Modulates Circulating Amino Acids

**Figure 4:**
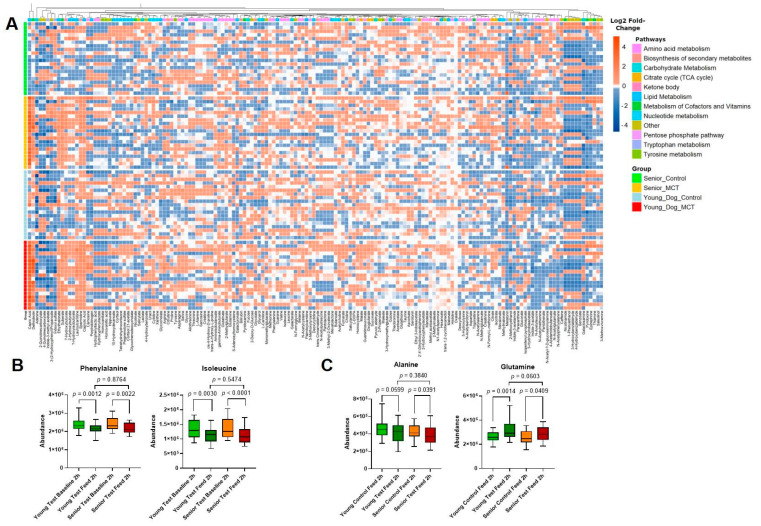
MCT feeding alters amino acid availability in the circulation. (**A**) Heatmap of polar metabolite changes in young adult and senior animals during control and MCT feeding. (**B**) Abundance of circulating aromatic amino acids, phenylalanine, and isoleucine in young adult and senior animals during control diet and MCT feeding. (**C**) Levels of glutamine and alanine in young adult and senior animals during control diet feeding and MCT diet feeding. Results from young adult (green) and senior (red) animals are shown and *p* statistics are from ANOVA with Šídák’s correction.

Additional analyses were performed to examine the impact of the MCT diet on circulating polar metabolites. Of the polar metabolites measured in senior dogs, 15 metabolites were significantly changed when comparing the control and MCT diets after feeding. In the young adult dogs (aged 1–6 years), 38 metabolites were significantly changed (Figure 4A).

The changes in polar metabolites were primarily mediated by differences in a subset of circulating amino acids When comparing the baseline samples to the 2 h post-feeding sample, animals fed the MCT diet, irrespective of age, showed a trend to decreased levels of amino acids, such isoleucine (a BCAA) and phenylalanine (an aromatic amino acid), while alanine was only decreased in senior animals (Figure 4A–C). When examining other amino acids, glutamine was increased with MCT feeding for both age groups.

## 4. Discussion

MCTs from the diet serve as efficient precursors for the generation of ketone bodies and have been investigated as nutritional solutions for disorders such as epilepsy [27,28], canine myxomatous mitral valve disease [29], and cognitive decline due to aging [4]. This study examined changes in the serum of both young adults and senior canines after the ingestion of a diet containing MCTs. Beyond evaluating ketones, metabolomics and lipidomics approaches enabled the evaluation of global metabolic alterations.

A unique feature of our study is the cross-over design that allowed us to assess individual canine responses to MCT supplementation according to age. The results show that, after five weeks of MCT diet, multiple lipid species from the diet (including MCTs, capric acids, and caprylic acid) accumulate in the circulation of both fasting and 2 h fed animals. We also observe increased levels of β-hydroxybutyrate, which is consistent with the rapid absorption of MCTs and subsequent oxidation for energy [30]. In addition to being used as an energy source, the results of this study show that dietary MCT supplementation also rewires lipid metabolism in canines. Multiple TG and diacylglycerol species were altered after MCT feeding. Some lipids, such as TG (46:2), increased, whereas others such as TG (16:0_17:0_18:1) decreased. In line with the observed alterations in TG composition, MCTs have been implicated in broader changes in lipid metabolism and storage [31]. For example, ingestion of MCTs have been shown to stimulate beta-oxidation of long-chain fatty acids [32]. Additionally, dietary MCTs increase lipolysis [33] and reduce white adipose tissue [34]. The results of this study also demonstrate that five weeks of dietary exposure to MCTs is sufficient to cause considerable changes in systemic lipid metabolism. It is well established that lipid metabolism changes as a function of aging [35]. Interestingly, we did not observe age-dependent responses to the MCT diet. These data indicate that MCT supplementation alters systemic lipid metabolism similarly in both young adult and senior dogs.

In addition to changes in lipid metabolism, MCT feeding also changed levels of circulating polar metabolites, although to a lesser extent than lipid metabolites. In both young and senior animals, the levels of phenylalanine and isoleucine were decreased after MCT feeding, the levels of glutamine were increased after MCT feeding, and alanine showed age-dependent changes in circulation. MCTs could impact circulating amino acids through multiple mechanisms. MCT dietary content has been shown to have a positive correlation with intestinal villus height [36] and alter the intestinal import of amino acids such as phenylalanine [37]. It is also interesting to note that type 2 diabetes is associated with increased postprandial concentrations of BCAAs and aromatic amino acids [38], and high branched-chain and aromatic amino acids precede the development of hyperglycemia in middle-aged men and women [39]. Baseline serum concentrations of phenylalanine were shown to be associated (positive hazard ratio) with type 2 diabetes in adults [40]. Additionally, both phenylalanine and isoleucine have been associated with an increased risk of incidence for type 2 diabetes [41]. The effect of dietary MCTs on postprandial phenylalanine and isoleucine suggests that dietary MCTs may improve metabolic health and reduce the risk of developing hyperglycemia and diabetes in people and animals. Further studies are needed to test this. Glutamine and alanine are non-essential amino acids, but play important roles in many cellular functions, including energy balance, immune function, and glucose metabolism [42]. Alterations in glutamine levels have been shown to be associated with a decreased risk for type 2 diabetes [41]. In contrast, elevated plasma alanine levels are correlated with hyperglycemia and an increased risk of type 2 diabetes [43,44]. In our study, MCTs decreased alanine levels and increased glutamine levels in senior animals. Therefore, dietary MCT-induced changes in postprandial blood amino acids levels may promote improved glucose management and metabolic health in senior dogs by reducing postprandial blood glucose spikes.

## 5. Conclusions

In summary, this study utilized metabolomics and lipidomics to track systemic changes in circulating small molecules after feeding canines an MCT-enriched diet. We find that dietary MCTs rewire both the metabolome and the lipidome of the serum, which has the capacity to influence organ function. While the impact of MCT supplementation has been investigated as a therapeutic option for diseases such as cognitive decline, the results of our work suggest that dietary MCTs may also have other beneficial effects on the general health of dogs. More work is needed to further investigate the mechanism(s) of action by which MCTs affect the metabolism of whole animals.

## Figures and Tables

**Table 1:** Beagle demographics for cross-over study of dietary MCT supplementation.

Age Group	Age Range (Years)	Sex		Body Weight (kg)	BCS
		Male	Female		
Senior	12.37 ± 0.58 Median: 12.40 Range: 7–15	6 (Neutered)	4 (Spayed)	12.49 ± 0.51	5.0
Young	3.40 ± 0.43 Median: 3.04 Range: 1–6	5 (3 Neutered)	5 (2 Spayed)	10.88 ± 0.46	5.0

**Table 2:** Labrador retriever demographics for cross-over study of dietary MCT supplementation.

Age Group	Age Range (Years)	Sex		Body Weight (kg)	BCS
		Male	Female		
Senior	9.93 ± 0.52 Median: 9.91 Range: 7–15	7 (6 Neutered)	3 (Spayed)	28.49 ± 0.93	5.1 ± 0.11
Young	4.08 ± 0.47 Median: 4.32 Range: 1–6	5 (2 Neutered)	4 (2 Spayed)	31.57 ± 1.00	5.6 ± 0.18

**Table 3:** Ingredients and chemical composition of diets.

	Control	MCT
Ingredients (%, as fed) *		
Plant protein sources **	61.71	61.81
Animal protein sources ^†^	30.28	30.21
Cereal grains ^‡^	44.80	44.82
Tallow	5.50	0.00
MCTs	0.00	5.50
Vitamins and minerals	1.67	1.67
Nutrient composition (% as fed) ***		
Moisture	8.02	8.29
Ash	6.39	6.34
Crude protein	30.23	29.8
Crude fat	14.50	14.4
Crude fiber	1.85	1.86
Energy content		
Calculated ME ^§^ (kcal/kg)	3620.07	3620.29

* Formulation values. ** Plant protein came from corn, barley, rice, wheat, corn gluten meal, and corn germ meal. ^†^ Animal protein came from chicken, dried eggs, and poultry byproduct meal. ^‡^ Cereal grains included corn, barley, rice, and wheat. *** Analytical values. ^§^ Calculated based on the predictive equation for metabolizable energy in dog foods [22].

**Table 4:** Composition and abundance of MCTs in feed samples and MCT oil.

Sample	MCT 8:0_8:0_8:0	MCT 8:0_8:0_10:0	MCT 8:0_10:0_10:0	MCT 10:0_10:0_10:0
Control diet	5178.00 ±3171.75	5272.67 ±2938.64	22,526.00 ±17,837.10	12,136.33 ±2710.77
Test diet	474,248.67 ±37,745.60	464,748.00 ±20,804.10	292,046.00 ±20,724.50	130,564.00 ±3698.66
MCT oil	1,065,087.33 ±13,388.60	903,900.00 ±21,984.10	756,068.00 ±15,604.10	318,508.33 ±19,009.20

## Data Availability

The raw data supporting the conclusions of this article will be made available by the authors upon request.

## Supplementary Materials

The following supporting information can be downloaded at: https://www.mdpi.com/article/10.3390/ani14243577/s1, Figure S1: β-hydroxybutyrate levels in beagles and Labrador retrievers after MCT feeding; Figure S2: Breed-specific changes in ketone bodies after MCT feeding.
