# Supplementary material for: Effects of Dietary Medium-Chain Triglyceride Supplementation on the Serum Metabolome of Young Adult and Senior Canines
Source: Animals (Basel). 2024 Dec 11;14(24):3577. doi: 10.3390/ani14243577 (PMC11672509; doi:10.3390/ani14243577)
Supplement: Supplementary file 1 [file animals-14-03577-s001.zip › animals-3288247-supplementary.pdf]

Supplementary Materials for

Effects of Dietary Medium-Chain Triglyceride Supplementation on the Serum Metabolome of Young Adult and Senior Canines

Yuanlong Pan [1,*], Miriam Sindelar [2,3,4], Ethan Stancliffe [2,3,4], Leah P. Shriver [2,3,4], Rondo P. Middleton [1] and Gary J. Patti [2,3,4,*]

1 Nestlé Purina Research, St. Louis, MO, 63164, USA; rondo.middleton@rd.nestle.com
2 Department of Chemistry, Washington University in St. Louis, St. Louis, MO, 63130, USA; miriam.sindelar@wustl.edu (M.S.); estancliffe@wustl.edu (E.S.); sleah@wustl.edu (L.P.S.)
3 Center for Mass Spectrometry and Metabolic Tracing, Washington University in St. Louis, St. Louis, MO, 63130 USA
4 Center for Human Nutrition, Department of Medicine, Washington University in St. Louis, St. Louis, MO, 63130, USA
* Correspondence: yuanlong.pan@rd.nestle.com (Y.P.); gjpattij@wustl.edu (G.J.P.)

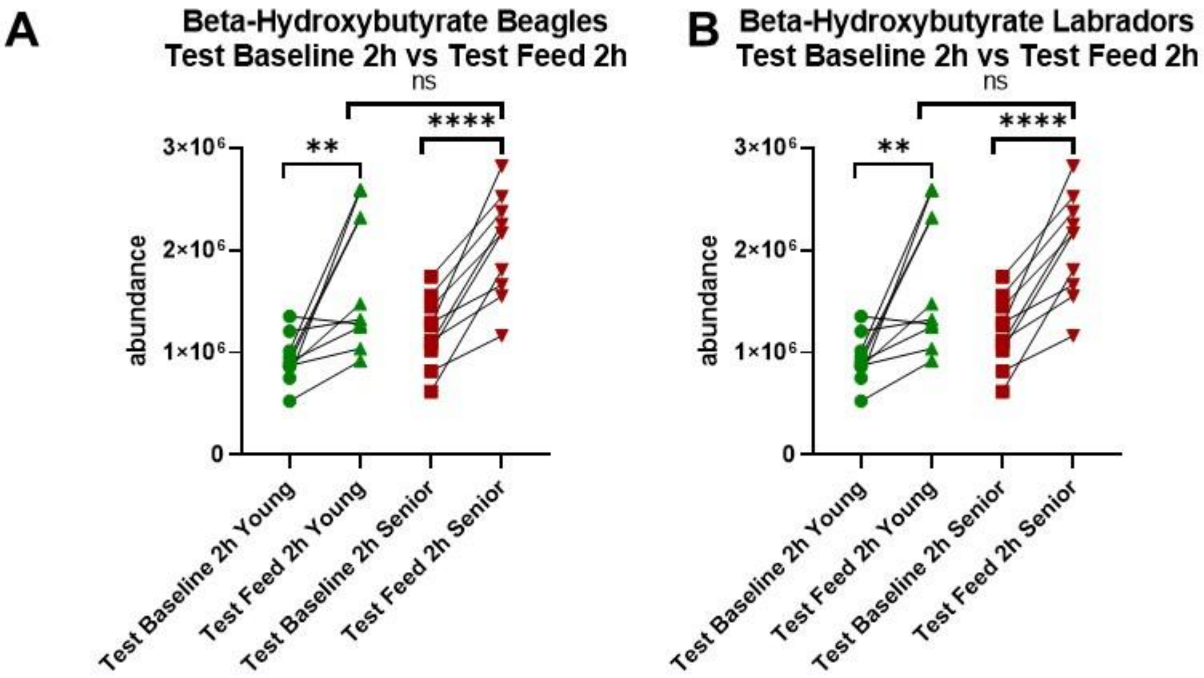

Supplementary Figure S1. β-hydroxybutyrate levels in beagles and Labrador retrievers after MCT feeding. Relative abundance of β-hydroxybutyrate in young adult and senior beagles (A) as well as young adult and senior Labrador retrievers after MCT feeding. Results from young adult (green) and senior (red) animals are shown and p statistics are from paired Student's t-tests.

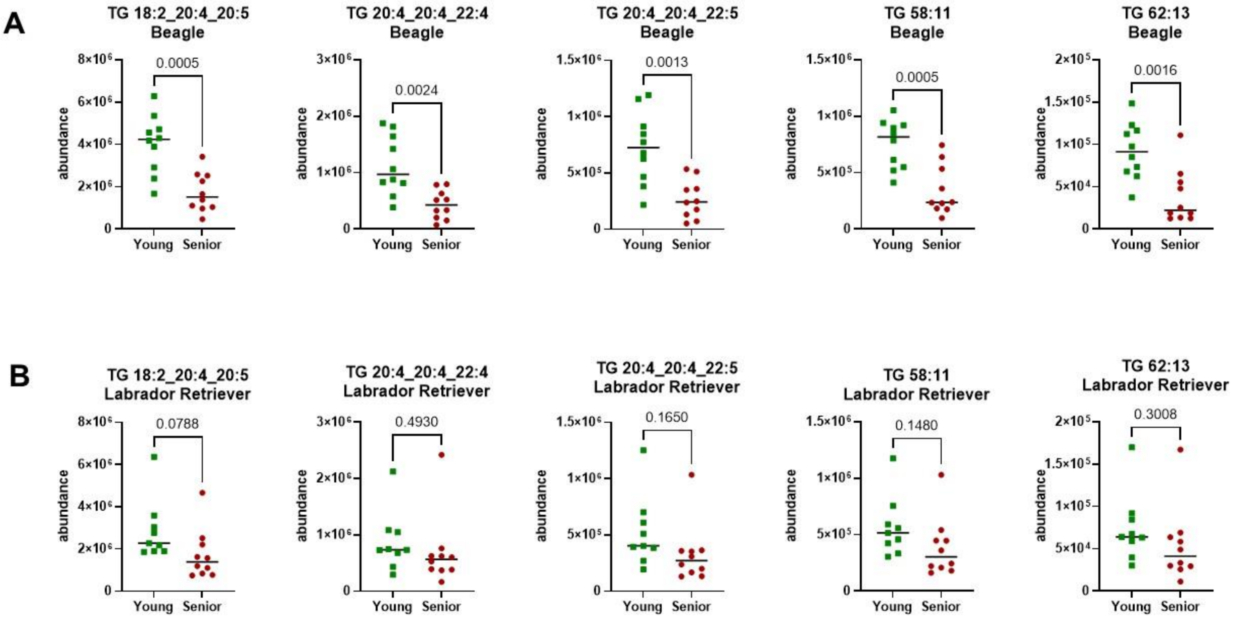

Supplementary Figure S2. Breed-specific changes in ketone bodies after MCT feeding. A) Relative abundance of TG species in beagles (A) and Labrador retrievers (B). Results from young adults (green) and senior (red) animals are shown and *p* statistics are from paired Student's t-tests.
